# Comparative Chloroplast Genomics and Codon Usage Bias Analysis in *Hevea* Genus

**DOI:** 10.3390/genes16020201

**Published:** 2025-02-06

**Authors:** Yang Yang, Xueyang Liu, Lixia He, Zhenhua Li, Boxuan Yuan, Fengyan Fang, Mei Wang, Aifang Li, Cheng Liu, Minmin He, Shugang Hui, Wenda Wang, Xuchu Wang

**Affiliations:** 1 Plant Stress Resistance Integrated Biology Laboratory, College of Life Sciences, Hainan Normal University, Haikou 571158, China; yy15776580499@163.com (Y.Y.); helixia0605@163.com (L.H.); 2 Key Laboratory of Plant Resource Conservation and Germplasm Innovation in Mountainous Region (Ministry of Education), Institute of Agro-Bioengineering, College of Life Sciences, Guizhou University, Guiyang 550025, China; yuanboxuan111@163.com (B.Y.); fangfengyan0913@163.com (F.F.); wangmei7102@163.com (M.W.); afangliguizhou@163.com (A.L.); hmmyia@163.com (M.H.); sghui@gzu.edu.cn (S.H.); 3 Photosynthesis Research Center, Key Laboratory of Photobiology, Institute of Botany, Chinese Academy of Sciences, Beijing 100093, China; liuxueyang@ibcas.ac.cn (X.L.); lizhenhua@ibcas.ac.cn (Z.L.); liucheng@ibcas.ac.cn (C.L.); 4 Academician Workstation of Agricultural High-Tech Industrial Area of the Yellow River Delta, National Center of Technology Innovation for Comprehensive Utilization of Saline-Alkali Land, Dongying 257300, China

**Keywords:** chloroplast genome, codon, RSCU, *Hevea*

## Abstract

Objectives: This study investigates the cpDNA sequences from six *Hevea* species, aiming to explore their genomic characteristics, gene content, and genetic relationships. The objectives include understanding the structure of these genomes, identifying potential gene rearrangements, and providing insights into genetic improvement and conservation strategies for the *Hevea* genus. Methods: cpDNA sequences from six *Hevea* species were sequenced and analyzed. Genome sizes, GC content, gene encoding potential, and structural integrity were assessed. Simple sequence repeats (SSRs) and codon usage were analyzed, with a focus on optimal codons and their frequency. Phylogenetic analysis was conducted to determine the genetic relationships within the *Hevea* genus. Results: The cpDNAs from the six species exhibited genome sizes ranging from 161,093 bp to 161,254 bp, with GC content between 35.72% and 35.75%. Each genome contained 91 to 92 protein-coding genes, with the infA gene consistently present. No significant gene rearrangements were detected, and SSR analysis revealed mono-repeats primarily composed of A/T bases. Codon usage analysis indicated that leucine is predominantly encoded by the UUA codon, and 31 optimal codons were identified, mainly ending in A or U. Phylogenetic analysis clarified the genetic relationships among the species. Conclusions: The study provides detailed insights into the cpDNA characteristics of *Hevea* species, highlighting stable genome structures, conserved genes, and specific patterns of codon usage. These findings are valuable for conservation efforts, genetic improvement strategies, and the sustainable use of *Hevea* germplasm.

## 1. Introduction

Natural rubber from rubber trees is a critical industrial raw material and strategic resource [1]. In December 2022, global rubber production reached 1.397 million tons. However, due to the enormous domestic consumption, China’s self-sufficiency rate for natural rubber is only 15% [2]. Given the current international situation and regional instability caused by the global debt crisis, addressing the supply issue of natural rubber is particularly urgent [3]. After a century of traditional breeding efforts by researchers both domestically and internationally, the rubber yield of wild populations has been significantly improved [4]. Nevertheless, due to climate and regional constraints, the suitable planting areas in China, including Hainan, Yunnan, and Guangdong province, have almost reached the limit [5].

Breeding cycles for woody plants such as rubber trees are prolonged, and their genetic composition is highly complex. Chloroplast genome (cpDNA) research provides valuable insights that can facilitate the development of crops with enhanced traits. Specifically, the study of cpDNA contributes to improving photosynthetic efficiency, understanding stress tolerance mechanisms, and identifying genes linked to desirable traits. Furthermore, chloroplast genome research plays a pivotal role in taxonomy, reconstructing phylogenetic relationships, and exploring the adaptive evolution of plant species. By 2024, 34,923 plant cpDNAs, including that of *Hevea brasiliensis* RRIM600 [6], had been sequenced and cataloged in the Chloroplast Genome Information Resource (CGIR) database. The *Hevea* genus consists of 11 species, with *H. brasiliensis* (RRIM600/PR107) [7], *Hevea pauciflora* [8], and *Hevea camargoana* [9] having their cpDNAs fully sequenced. Various *Hevea* varieties are extensively cultivated in China’s rubber-growing regions, contributing significantly to socio-economic benefits and fostering the rapid and sustainable growth of the natural rubber industry [10]. This research aims to investigate the variations in gene content, intra-species diversity, and structural differences in the cpDNAs among different *Hevea* genus. Our findings highlight the importance of chloroplast genome data in understanding the evolution, phylogeny, and genetic diversity of the *Hevea* genus. This information provides valuable insights into the genetic resources of *Hevea* species, offering a theoretical foundation for future conservation, genetic improvement, and breeding programs.

## 2. Materials and Methods

### 2.1. Sequence Retrieval and Filtering

The cpDNAs of six *Hevea* species were downloaded from the NCBI GenBank database (Table 1) [11]. Utilizing Geneious 9.0.2 software [12], we performed statistical and comparative analyses on gene composition and structure, focusing on GC content and sequence lengths of LSC, IRb, SSC, and IRa regions. Data analysis and visualization were carried out using Excel 2019 and OriginPro 2021 [13].

### 2.2. IR/SC Boundary Contraction and Expansion Analysis

Using IRscope [14], a comparative study of IR/SC boundaries in the cpDNAs of *Hevea* species was conducted, creating diagrams to illustrate IR/SC region boundaries. We examined the differences at the four junction sites of chloroplast genome regions. For the analysis of six *Hevea* cpDNAs, *H. brasiliensis* served as the reference sequence [15]. All sequences were aligned using MAFFT v7.450 software [16]. Post-alignment, manual inspection, and corrections were performed to ensure data precision. Collinearity analysis was conducted on the aligned sequences using the Mauve tool function in Geneious 9.0.2 software to deepen our understanding of the genomic structural relationships among different *Hevea* species. A Python script extracted annotation information from chloroplast genome gb files, and along with the genome sequences, these were uploaded to the mVISTA platform [17], which was used for comprehensive genome comparison to detect sequence variations. Initially, genome comparison diagrams were generated using mVISTA.

### 2.3. SSR Analysis

SSRs known for their significant polymorphisms [18] were analyzed, using the MISA-web tool [19] to determine the base sequences at SSR locations. REPuter was employed to examine types of repeat sequences [20]. The analysis parameters included a minimum match score of 50, and a maximum of 3 mismatches for the analysis of dispersed repeat sequences.

### 2.4. Phylogenetic Study of Hevea Chloroplast DNA

The chloroplast DNA sequences of six *Hevea* species were retrieved from the NCBI database, as detailed in Table 1. To ensure the accuracy and consistency of the sequences, we performed a BLAST analysis to validate their similarity and quality before subsequent analyses. Using Geneious 9.0.2 software, we constructed a phylogenetic tree to determine the evolutionary relationships and phylogenetic positions of the six *Hevea* species. This approach allowed us to confirm the reliability of the sequences and ensured robust phylogenetic analysis.

### 2.5. Analysis of Codon Usage Frequency

CodonW 1.4.2 software was used for statistical analysis of codon usage [21]. The EMBOSS platform analyzed the GC content of coding sequences (CDS) (designated as GC1, GC2, and GC3, respectively) and their averages [22]. Correlation studies were conducted using R 4.3.1. Pearson correlation coefficients were calculated using the cor function in base R, and significance testing was performed with cor.test. Results were visualized using the ggcorrplot package 0.1.4.1 [23]. The selection criteria for CDSs in *Hevea* cpDNAs were as follows: (1) CDSs must have a base count that is a multiple of three for sequence completeness. (2) The minimum coding sequence length should be 300 bp to ensure reliability. (3) High-quality sequences should contain only standard bases, avoiding non-standard bases. (4) CDSs should be free of internal stop codons to ensure proper transcription. Perl scripts were employed to filter CDSs according to these rules.

### 2.6. Analysis of RSCU and Its Frequency

Relative synonymous codon usage (RSCU) is a key metric for measuring codon usage bias in gene expression [24]. The formula is as follows:

$$RSCU=\frac{x_{ij}}{\sum_{j}^{n_{i}}x_{ij}}n_{i}$$

### 2.7. ENc-Plot Analysis

The effective number of codons (ENc) measures codon usage bias, with values ranging from 20 to 61 [25]. The GC3s value represents the ratio of G and C content at the third position of a codon (excluding Met and Trp) to the total number of bases in the gene [26]. The expected ENc value is calculated using

$$ENc=2+S+\frac{29}{S^{2}+{\left(1-S\right)}^{2}}$$

Research on optimal codons depends on ENc bias. CDSs are sorted by their ENc values, and the highest and lowest 10% of genes are chosen to form a biased gene library [27]. Codons with a ΔRSCU value ≥ 0.08 and an RSCU value > 1 are considered high-frequency codons [28]. Codons meeting both conditions are identified as optimal codons.

The PR2-plot method is employed to assess the base composition at the third position of codons in amino acids [29]. The extent to which the bases cluster toward the center (where A equals T and C equals G) and their distribution direction within the plot highlight base preferences.

Neutrality analysis of GC12 and GC3 evaluates the effects of mutations and natural selection on codon usage [29]. Correspondence analysis (COA) based on RSCU was utilized to compare codon usage patterns, excluding codons for Met, Trp, and the three stop codons. This method represented the variation in codon usage through orthogonal axes, identifying the primary sources of gene variation [30].

### 2.8. Photosystem Protein Structure Prediction and Functional Annotation

The protein sequences of Photosystem I (PSI) and Photosystem II (PSII) in *H. brasiliensis* were used for three-dimensional (3D) structure modeling and functional prediction via SWISS-MODEL. The core subunits of PSI and PSII (e.g., PsaA, PsaB, PsbA, PsbD) were identified through sequence alignment and were annotated to their functional roles within the photosystems. Additionally, chloroplast proteomic data were obtained using mass spectrometry to quantify the protein abundance of PSI and PSII. For this analysis, chloroplast proteins were extracted from *H. brasiliensis* leaves and digested with trypsin. Protein identification and quantification were performed using an AB SCIEX TripleTOF 5600 mass spectrometer in positive ion mode. The system was operated with a mass range of 350–1800 *m*/*z* for MS and 100–1500 *m*/*z* for MS/MS. The raw data were processed using ProteinPilot software (version 5.0), and a false discovery rate (FDR) of 1% was applied for peptide and protein identification. Label-free quantification was conducted to determine relative protein abundances. Heatmap visualization of protein abundance differences was generated using the R package pheatmap 1.0.12, providing a comparative analysis of PSI and PSII subunit abundance across samples. These results offer valuable insights into the functional roles and differential expression of chloroplast proteins in *H. brasiliensis*.

## 3. Results

### 3.1. Structural Attributes of the Hevea Chloroplast Genome

The analysis revealed that the lengths of the *Hevea* cpDNAs span from 161,093 bp (*H. spruceana*) to 161,254 bp (*H. camargoana*). The LSC, SSC, IRA, and IRB regions’ lengths ranged from 89,079 to 89,281 bp, 18,362 to 18,376 bp, 26,810 to 26,819 bp, and 26,810 to 26,819 bp, respectively. The GC content in these genomes surpasses 15%, varying from 35.72% to 35.75%. The gene counts for rRNA and tRNA were notably consistent, with eight rRNA genes and 36 to 37 tRNA genes. The chloroplast genome comprised 91 to 92 genes coding for proteins. Within the IR region, there were eight tRNA genes, four rRNA genes, and seven genes (rps19, rpl2, ycf2, rpl23, ndhB, rps7, and rps12). Importantly, a distinctive gene, infA, was identified across all *Hevea* species. The *Hevea* cpDNAs were annotated with 135 to 137 genes, which included 91 to 92 protein-coding genes (Table 1).

### 3.2. Comparative Genomic Analysis

**Figure 1:**
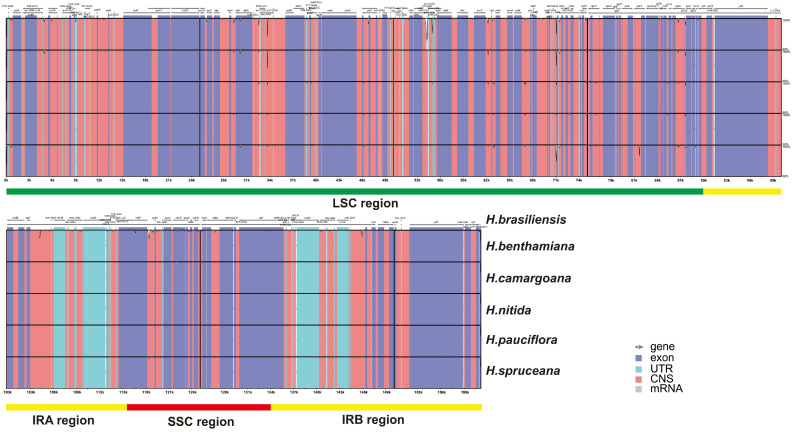
Visual representation of the *Hevea* chloroplast genome utilizing mVISTA. Note: In the diagram, gene orientations are denoted by gray arrows.

The cpDNAs of the *Hevea* genus show highly similar sequences. While the coding regions of these genomes are relatively consistent, the non-coding regions exhibit greater variability. Highly divergent areas are primarily those such as ndhK-atpE, accD-PsaI, PetG-PsaJ, rps7-trnV-GAC, and ndhF-rpl32 (Figure 1). These divergent areas may be used for phylogenetic studies or to differentiate among the six cpDNAs. Examination of potential rearrangement events showed that the genome structures and gene sequences are largely conserved, with no observed gene rearrangements. Using the Mauve method, the six *Hevea* cpDNAs were aligned with *H. brasiliensis* as the reference. High consistency was observed among these genomes. Whole-genome alignment results indicated no rearrangements or inversions, maintaining complete collinearity among the six genomes. Among the four components of the cpDNAs, the IR region exhibited the least sequence variation, while the LSC region showed the most. Variations in genome length and gene number may result in the absence of certain genes in specific species.

### 3.3. IR Region Contraction and Expansion

**Figure 2:**
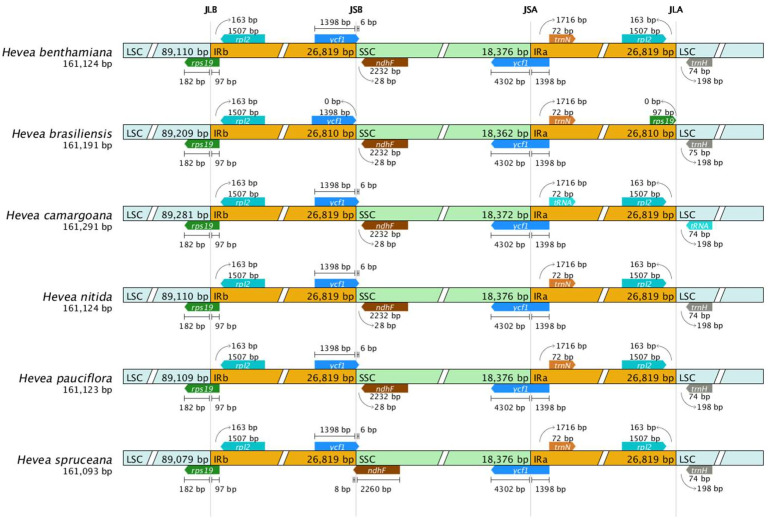
Comparison of the boundaries of LSC, SSC, and IR regions in the *Hevea* cpDNAs. Note: Genes are depicted by short bars, and the intervals and boundaries between genes are represented by base pair length. Gene extensions are indicated above the bars.

Among the six *Hevea* cpDNAs, *H. camargoana* has the largest chloroplast genome (161,291 bp) with an IR of 53,638 bp, while *H. spruceana* has the smallest chloroplast genome (161,093 bp). By examining the IR/SSC junctions in the *Hevea* genus, we discovered distinct patterns and trends in different species, indicating that these regions might have adaptive roles during evolution (Figure 2).

### 3.4. Analysis of Long Repeats and SSRs

**Figure 3:**
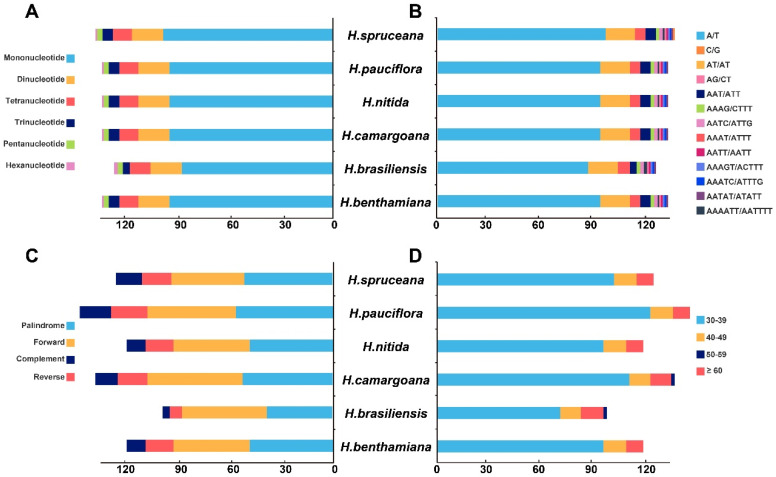
Quantitative analysis of SSRs and long repeat sequences in the *Hevea* genus chloroplast genome: (**A**) types of SSRs, (**B**) frequency of identified SSRs, (**C**) types of long repeat sequences, and (**D**) number of long repeat sequences by length.

Six types of simple sequence repeats (SSRs) were identified in the cpDNAs of *Hevea* species. The number of cpSSRs ranged from 126 in *H. brasiliensis* to 137 in *H. spruceana*. Mononucleotide repeats were the most prevalent, representing 69.05% (*H. brasiliensis*) to 71.53% (*H. spruceana*) of the total SSRs, followed by dinucleotide repeats (13.14% to 14.29%) and trinucleotide repeats (3.17% to 4.51%). The percentage of tetranucleotide repeats, varying from 8.03% to 9.52%, was higher than that of trinucleotide (3.17% to 4.51%) and pentanucleotide repeats (1.50% to 2.38%). Pentanucleotide and hexanucleotide repeats were found in all six cpDNAs analyzed, with hexanucleotide repeats being particularly uncommon, comprising only 0.73% to 1.59% (Figure 3). Most SSRs consisted of A/T repeats rather than G/C repeats. The count of these repeats varied from 98 in *H. brasiliensis* to 146 in *H. pauciflora*. Despite notable differences in the lengths of forward and palindromic repeats, many interspersed repeats were between 30 to 39 bp in length (Figure 3).

### 3.5. Codon Usage Bias Analysis

**Figure 4:**
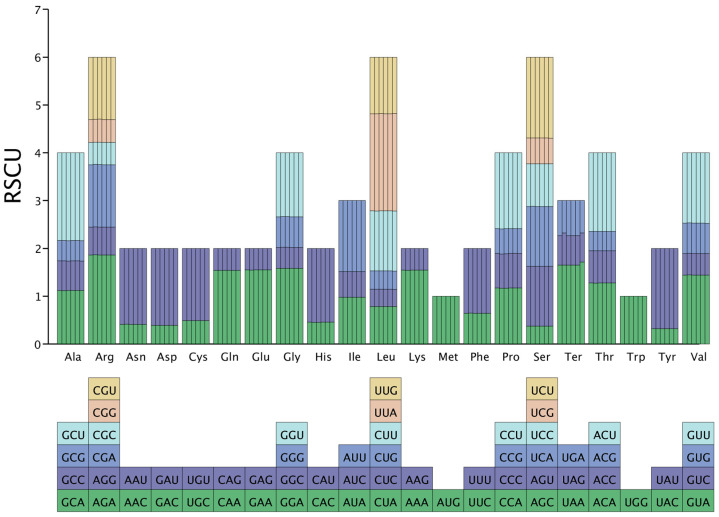
Relative usage of synonymous codons in the *Hevea* cpDNAs.

In the analysis of *Hevea* chloroplast coding sequences, leucine (Leu) was predominantly encoded by the UUA codon, exhibiting a high RSCU value exceeding 2.00 (2.0291–2.0376). Conversely, tyrosine (Tyr) was encoded to a much lesser extent, with an RSCU value between 0.3217 and 0.3243. Met and Trp had RSCU values precisely at 1.00 (Figure 4).

### 3.6. Analysis of ENc-Plot

**Figure 5:**
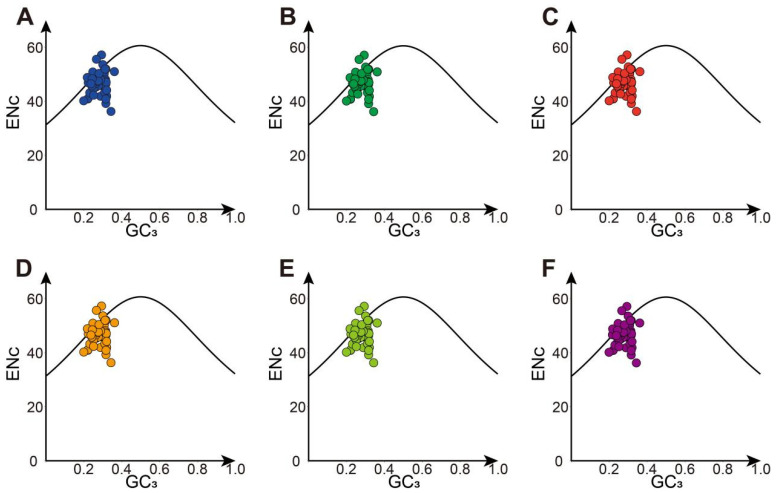
The relationship between the ENc and GC3 in the chloroplast genome of *Hevea* genus (ENc-plot). (**A**) *H. benthamiana*; (**B**) *H. brasiliensis*; (**C**) *H. camargoana*; (**D**) *H. nitida*; (**E**) *H. pauciflora*; (**F**) *H. spruceana*.

In this study, the average ENc for cpDNAs ranged from 46.47 to 46.48, with individual values spanning from 35.87 to 56.8. The genes with ENc values above 45 numbered between 43 and 45. Many *Hevea* chloroplast genes fell below the anticipated ENc standard curve, indicating that natural selection predominantly shapes codon usage bias (Figure 5). ENc-plot analysis shows that the ENc values matched the predicted values, suggesting that codon choice was driven mainly by base variations.

### 3.7. Analysis of PR2-Bias Plot

**Figure 6:**
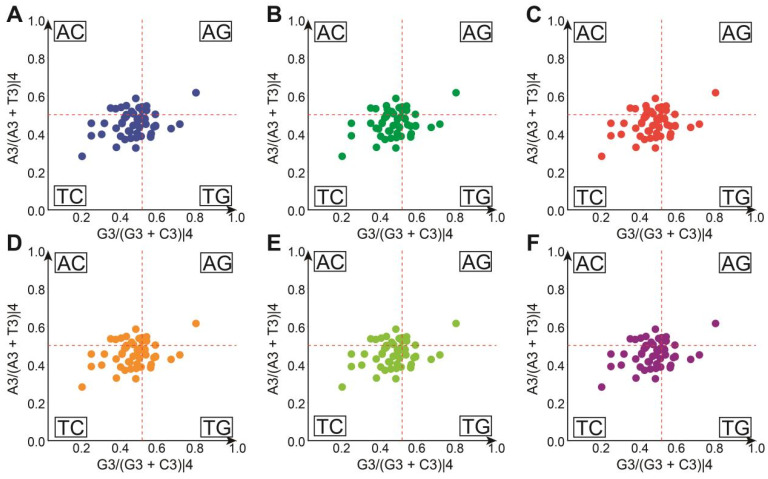
Codon bias analysis of *Hevea* chloroplast genome (PR2-plot). (**A**) *H. benthamiana*; (**B**) *H. brasiliensis*; (**C**) *H. camargoana*; (**D**) *H. nitida*; (**E**) *H. pauciflora*; (**F**) *H. spruceana*.

The overall average GC content in *Hevea* cpDNAs ranges from 37.64% to 37.80%. Specifically, GC1, GC2, and GC3 range from 28.02% to 45.32%, 26.16% to 56.12%, and 19.77% to 36.15%, respectively. These values indicate a preference for A/U bases over G/C bases in the codon selection of *Hevea* cpDNAs. Additionally, the varying GC content at GC1, GC2, and GC3 underscores the differences among codon positions (Figure 6).

When examining the six *Hevea* chloroplast genome data sets, their coordinate points were unevenly spread across the four quadrants of the graph, primarily concentrated in the G3/(G3+C3) < 0.5 and A3/(A3+T3) < 0.5 regions. This suggests a higher frequency of C relative to G at the third codon position, and T being used more frequently than A. If base mutation were the sole factor in codon usage preference, the frequencies of A/T and G/C would be expected to be equal or nearly equal. PR2-plot analysis indicates that codon usage bias in *Hevea* cpDNAs is a complex process driven by the interaction of base mutations and natural selection.

### 3.8. Analysis of Neutrality Plot

**Figure 7:**
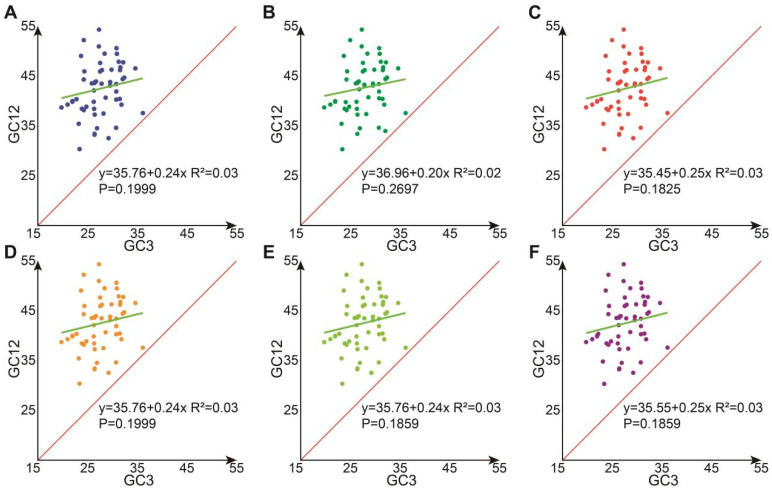
Codon neutrality mapping analysis of *Hevea* chloroplast genes. (**A**) *H. benthamiana*; (**B**) *H. brasiliensis*; (**C**) *H. camargoana*; (**D**) *H. nitida*; (**E**) *H. pauciflora*; (**F**) *H. spruceana*.

The neutrality plot analysis of *Hevea* chloroplast genes shows that these genes are relatively concentrated, all positioned above the diagonal line. The GC12 values range from 30.32% to 54.32%, while GC3 values range from 19.77% to 36.15%. The correlation coefficients between GC12 and GC3 span from 0.1825 to 0.2697, suggesting that mutational pressure does not significantly impact codon usage bias. Additionally, all points are above the 1:1 line, indicating that GC12 is greater than GC3, which implies that the G+C content at the third codon position is low (Figure 7). The regression slope analysis indicates that the influence of mutational pressure on codon usage patterns in cpDNAs ranges from 20.00% to 25.00%, indicating that mutational pressure has a relatively minor influence, while natural selection is the main factor driving codon selection preference.

### 3.9. Analysis of Correspondence

**Figure 8:**
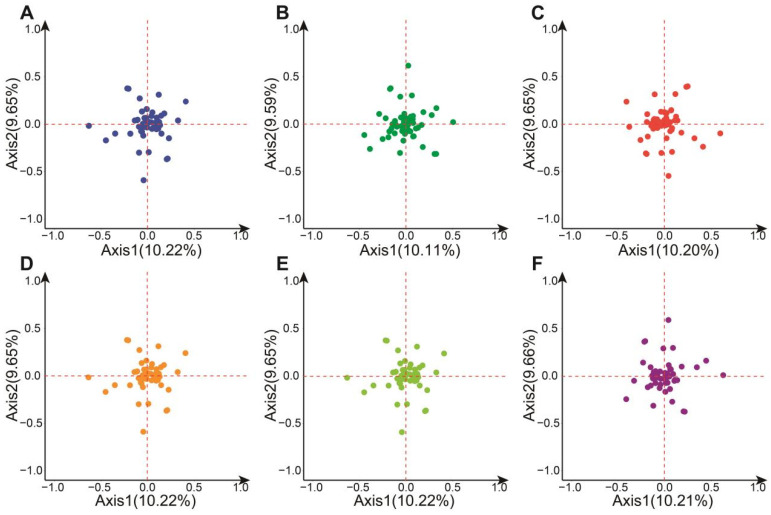
COA of *Hevea* chloroplast genome. (**A**) *H. benthamiana*; (**B**) *H. brasiliensis*; (**C**) *H. camargoana*; (**D**) *H. nitida*; (**E**) *H. pauciflora*; (**F**) *H. spruceana*.

Using COA based on RSCU, the RSCU of each gene was plotted against the average values on axes 1 and 2 at the origin. The genes were distributed throughout the coordinate system of axes 1 and 2, showing no significant preference, which indicates a certain uniformity in codon usage among different chloroplast genes. Further examination of codon diversity sources in the *Hevea* chloroplast genome positioned the coding sequences (CDSs) on the principal component analysis plane. COA revealed that the contribution rates of axes 1 and 2 range from 10.11% to 10.22% and 9.59% to 9.66%, respectively (Figure 8). This finding indicates that the first axis contributes the most to explaining codon usage preference but only accounts for part of the variation. Genes were grouped into five functional categories based on function: genetic system genes, ribosomal protein genes, photosystem genes, other protein genes, and conserved open reading frames. The dispersed distribution of different categories of genes on the COA plane indicates significant differences in codon usage patterns among genes with various functions. The development of codon usage bias in *Hevea* chloroplast genes is a complex process influenced by multiple factors, not just a single factor. Although the first axis plays the largest role in revealing codon usage bias, it only represents a portion of the variation, suggesting the existence of other influencing factors not accounted for.

**Figure 9:**
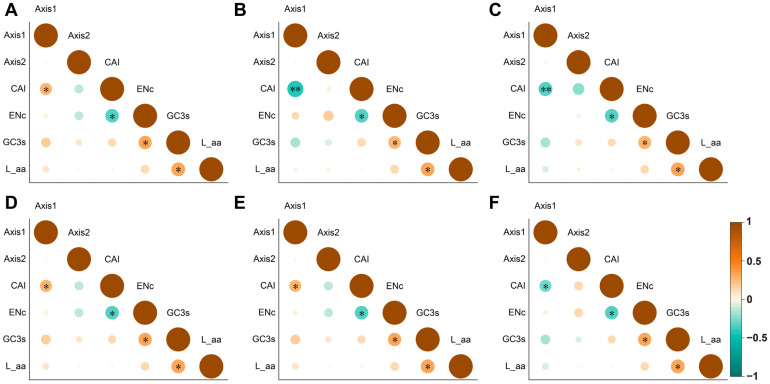
COA of axis 1, axis 2, and codon usage indices of the cpDNAs in six *Hevea* species. (**A**) *H. benthamiana*; (**B**) *H. brasiliensis*; (**C**) *H. camargoana*; (**D**) *H. nitida*; (**E**) *H. pauciflora*; (**F**) *H. spruceana*. * Indicates *p* < 0.05, ** indicates *p* < 0.01.

Further codon usage analysis demonstrated that ENc and CAI were negatively correlated with axis 1, while GC3 content showed a positive correlation with ENc (Figure 9). The same species show a negative correlation between CAI and axis 1, indicating lower CAI may reduce gene expression efficiency due to specific environmental pressures. Additionally, in *H. benthamiana*, *H. nitida*, and *H. pauciflora*, this negative correlation highlights the inhibition of codon adaptation and diversity. Conversely, a positive correlation between CAI and axis 1 in *H. brasiliensis*, *H. camargoana*, and *H. spruceana* indicates different regulatory patterns. Overall, ENc and CAI are negatively correlated with axis 1, while GC3 is positively correlated with ENc and L_aa (Figure 9). These patterns suggest that gene groups enhance adaptability through multiple factors, reflecting different strategies to adjust gene expression and adapt to environmental and evolutionary pressures.

### 3.10. Optimal Codons

**Figure 10:**
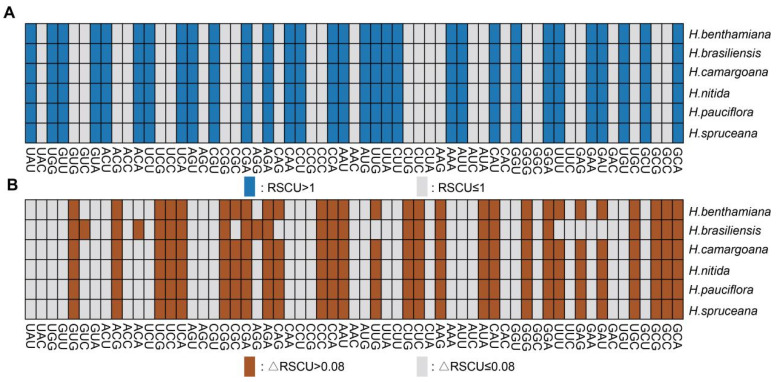
RSCU (**A**) and ΔRSCU (**B**) of six *Hevea* species.

Further examination of the coding sequences of *Hevea* chloroplasts revealed 30 codons with an RSCU value not exceeding 1.00, most of which (29) end in G or C. In contrast, 29 codons have an RSCU value greater than 1.00, most of which (28) end in A or U. These findings clearly indicate a higher preference for codons ending in A or U in *Hevea* cpDNAs compared to those ending in G or C (Figure 10A). Based on the criterion of RSCU > 1, 30 high-frequency codons were identified in *Hevea* species, with 48.28% ending in U, 48.28% in A, and only one ending in G. These data emphasize a significant preference for codons ending in U/A in the *Hevea* chloroplast genome (Table S1). A total of 31 optimal codons were identified based on RSCU > 1 and ΔRSCU ≥ 0.08, with 22 conserved across all six species. In the *Hevea* genus, 31 optimal codons were identified. Among the six *Hevea* cpDNAs, 22 common codons were identified, including Ala (GCA), Ala (GCC), Ala (GCG), Cys (UGC), Gly (GGA), Gly (GGG), His (CAU), Ile (AUA), Lys (AAG), Leu (CUC), Leu (CUG), Asn (AAU), Pro (CCA), Pro (CCC), Arg (AGA), Arg (CGA), Arg (CGG), Ser (UCA), Ser (UCC), Ser (UCG), Thr (ACG), and Val (GUG). Among these, Ser (UCA), Ser (UCG), Asn (AAU), Ala (GCC), Gly (GGA), Val (GUG), Leu (CUG), Cys (UGC), Gly (GGG), Arg (AGA), and Pro (CCC) have ΔRSCU ≥ 0.3, while Leu (CUG), Cys (UGC), Gly (GGG), Arg (AGA), and Pro (CCC) have ΔRSCU ≥ 0.5 (Figure 10B, Table S2).

### 3.11. Phylogenetic Analysis of Hevea cpDNAs

**Figure 11:**
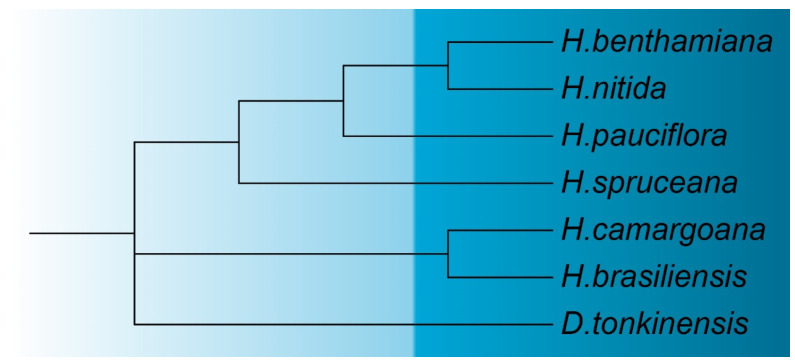
Phylogenetic analysis of the cpDNAs of the *Hevea* genus.

A detailed analysis of the six complete chloroplast genome datasets was performed to construct a phylogenetic tree of the *Hevea* genus. *Deutzianthus tonkinensis*, another member of the *Euphorbiaceae* family, was utilized as an outgroup to build the phylogenetic tree alongside the cpDNAs of the six *Hevea* species. *H. brasiliensis* and *H. camargoana* showed relatively close genetic relationships. In another clade, *H. spruceana*, *H. benthamiana*, *H. nitida*, and *H. pauciflora* formed a closely related group (Figure 11). Constructing this phylogenetic tree not only enhanced our understanding of the genetic diversity and relationships within the *Hevea* genus but also provided essential scientific evidence for future conservation, genetic improvement, and sustainable use of germplasm resources.

### 3.12. Structural and Quantitative Analysis of Photosystem Proteins

**Figure 12:**
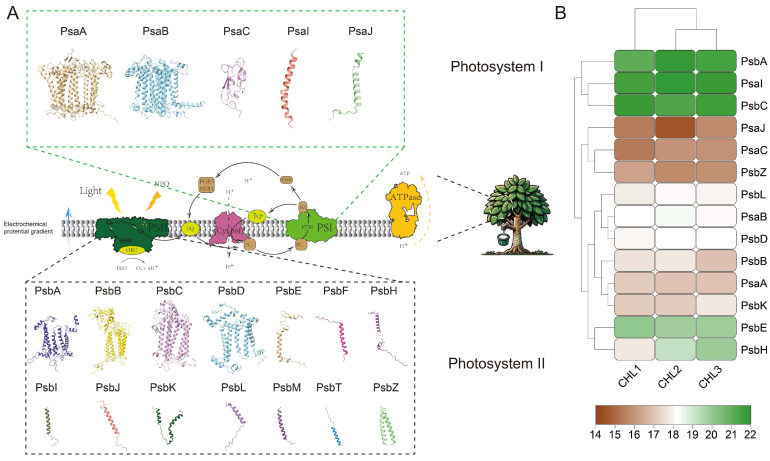
Three-dimensional (3D) Structure Prediction and Abundance Analysis of PSI and PSII Proteins in *H. brasiliensis*. (**A**) The 3D structures of core proteins from PSI and PSII were predicted using SWISS-MODEL. (**B**) Heatmap of protein abundance levels for major subunits of PSI and PSII based on chloroplast proteomic data. Different colors indicate variations in protein abundance, with green representing high abundance and brown representing low abundance.

Figure 12A illustrates the predicted three-dimensional (3D) structures of Photosystem I (PSI) and Photosystem II (PSII) proteins in *H. brasiliensis*, based on SWISS-MODEL structural modeling. The core subunits of PSI (e.g., PsaA, PsaB, PsaC) and PSII (e.g., PsbA, PsbD, PsbC) were successfully identified, alongside auxiliary subunits such as PsaI (PSI) and PsbE (PSII). This analysis highlights the high level of conservation of photosystem protein structures in *H. brasiliensis*, which is consistent with observations in other plant species. Moreover, the conserved nature of PSI and PSII subunits suggests that the evolutionary dynamics of the chloroplast genome have preserved essential genes for photosynthetic function, maintaining their structural integrity and functional relevance. Figure 12B provides a quantitative analysis of the protein abundance of PSI and PSII subunits based on chloroplast proteomic data, visualized through a heatmap. The data reveal that core PSI subunits, particularly PsaA and PsaB, exhibit the highest abundance, underscoring their crucial roles in light energy capture and electron transfer. Similarly, PSII subunits PsbA and PsbD, integral to oxygen-evolving activity and electron transfer, also show consistently high abundance across chloroplast samples. These findings align with the transcriptional and translational priorities encoded in the *H. brasiliensis* chloroplast genome, where genes for core photosynthetic components (e.g., psaA, psaB, psbA, and psbD) are not only highly conserved but also encoded directly in the chloroplast genome to ensure efficient regulation and expression. Notably, the analysis suggests potential interplay between the chloroplast genome and nuclear genome in regulating photosystem assembly. While core subunits are encoded by the chloroplast genome, many auxiliary proteins and regulatory factors are encoded by the nuclear genome and imported into the chloroplast. This division of genetic responsibility reflects the co-evolution of the chloroplast and nuclear genomes to optimize photosynthetic efficiency. Furthermore, the protein abundance patterns suggest differential regulatory mechanisms between PSI and PSII, possibly reflecting distinct roles in light harvesting under varying environmental conditions. In conclusion, this integrated analysis of chloroplast proteomics and structural prediction underscores the critical roles of PSI and PSII subunits in *H. brasiliensis*, as well as the evolutionary and functional significance of the chloroplast genome in sustaining photosynthetic efficiency. Future studies may focus on how environmental stressors influence the expression and abundance of these subunits, providing further insights into the adaptive mechanisms of chloroplast function.

## 4. Discussion

CpDNAs within the *Hevea* genus display remarkable sequence similarity, with internal repeat regions exhibiting higher conservation compared to single-copy regions. This level of conservation is vital for preserving genomic stability and ensuring proper chloroplast functionality [31]. Similar observations have been made in other plant genera, such as the Poaceae, where conserved repeat regions are essential for chloroplast genome stability [32]. Although coding regions are relatively consistent, non-coding regions show greater variability [33]. For example, in the *Brassicaceae*, the trnH-psbA intergenic spacer is frequently employed in phylogenetic studies due to its variability [34]. Our findings, which show no gene rearrangements and complete collinearity among the six genomes, are consistent with previous research highlighting the stability of cpDNAs in plants [33]. Research on the *Fabaceae* family supports this stability, with minimal chloroplast genome rearrangements observed [35]. Our data suggest that the GC content of *Hevea* cpDNAs exceeds 35%, with genome lengths varying from 161,093 bp (*H. spruceana*) to 161,254 bp (*H. camargoana*). The lengths of the LSC, SSC, IRA, and IRB regions show slight variation among species. The overall GC content, ranging from 35.72% to 35.75%, aligns with trends seen in other plant species, where GC content is critical for genome stability and function [32,36]. In the *Asteraceae* family, similar GC content trends correlate with genome stability [37]. The highly conserved sequences, with 91 to 92 protein-coding genes, further underscores the evolutionary stability of *Hevea* cpDNAs. This finding is consistent with the *Solanaceae* family, where conserved gene numbers are essential for chloroplast functionality [38].

Our research indicates that *H. camargoana* has the longest chloroplast genome, while *H. spruceana* has the shortest. The patterns of IR region expansion and contraction among *Hevea* species suggest adaptive functions during their evolution [39]. Similar patterns have been observed in the *Orchidaceae* family, where IR expansion and contraction significantly impact genome size [40]. Expanding IR regions can enhance genome stability and increase gene numbers, contributing to functional expression and regulatory mechanisms in chloroplasts [41]. However, excessive IR expansion can lead to genomic instability, warranting further research to understand its specific impacts on *Hevea*’s physiological functions [42]. Our analysis identified six types of SSRs in *Hevea* cpDNAs, with cpSSRs numbers ranging from 126 to 137. Mononucleotide repeats are the most prevalent, consistent with findings in other plant species where SSRs are crucial for genetic diversity and adaptability [31]. For instance, in the *Rosaceae* family, SSRs have been identified as key markers for genetic diversity studies [43]. The predominance of A/T repeats over G/C repeats aligns with the high AT content observed in *Hevea* cpDNAs and other plants [33]. This trend is also seen in the Fabaceae family, where high AT content is associated with repeat regions [35].

*Hevea* cpDNAs exhibit a marked preference for codons ending in A or U, with Leu predominantly encoded by the UUA codon. This A/U-ending codon bias is prevalent in cpDNAs and can improve translational efficiency [36,44]. In the *Poaceae* family, a similar bias toward A/U-ending codons has been noted, enhancing protein synthesis efficiency [45]. ENc-plot analysis indicates that natural selection significantly influences codon usage bias, with some genes showing weak bias influenced by base variations [46,47]. This bias is likely an adaptive mechanism to optimize protein synthesis within the chloroplast’s environment. In the *Asteraceae* family, similar adaptive mechanisms for codon usage bias have been proposed [37]. PR2-bias plot analysis of *Hevea* cpDNAs shows a preference for T/C at the third codon position, suggesting that codon selection patterns are influenced by factors beyond mutational pressure, such as natural selection [36]. Neutrality plot underscores the complex interplay between mutational pressure and natural selection in shaping codon usage patterns in *Hevea* cpDNAs. COA based on RSCU values indicates significant differences in codon usage patterns among *Hevea* chloroplast genes with varying functions. This dispersed distribution suggests that codon usage bias is influenced by multiple factors [46]. The identification of 31 optimal codons, most ending in A or U, further highlights the preference for these codons in *Hevea* cpDNAs. The identification of 31 optimal codons, primarily ending in A or U, provides a valuable resource for genetic engineering. Optimizing gene expression in the transgenic *Hevea* genus could be achieved by selecting codons with high translational efficiency, thereby enhancing protein synthesis in chloroplasts.

Our phylogenetic analysis of *Hevea* cpDNAs reveals a close genetic relationship between *H. brasiliensis* and *H. camargoana*, while *H. spruceana*, *H. benthamiana*, *H. nitida*, and *H. pauciflora* form another closely related cluster. This phylogenetic arrangement offers critical insights into the genetic diversity and evolutionary relationships within the *Hevea* genus, aiding future conservation and genetic enhancement initiatives [7]. Similar phylogenetic structures are observed in the *Cucurbitaceae* family, where close genetic relationships guide conservation strategies [48]. This research lays a theoretical groundwork for genomic studies and chloroplast genetic engineering in *Hevea*. By examining gene variations and codon usage patterns, we have deepened our understanding of photosynthesis characteristics and adaptability in *Hevea* species, particularly in low-temperature environments [49]. Future studies should investigate the functional implications of specific gene variations and explore the potential of genetic engineering to introduce advantageous traits into *Hevea* cpDNAs, utilizing advanced genomic and transcriptomic techniques [50]. Similar methods have been effectively used in the Solanaceae family to enhance crop resilience and productivity [51].

## 5. Conclusions

We performed comparative genomic and phylogenetic analyses of chloroplasts in the *Hevea* genus. The chloroplast genome sizes of *Hevea* species range between 161,093 bp and 161,254 bp, featuring a typical four-part structure: the LSC ranging from 89,079 to 89,281 bp, the SSC spanning 18,362 to 18,376 bp, and two IRs measuring between 26,810 and 26,819 bp. A total of 135 to 137 genes were identified. The chloroplast sequences show high conservation, with significant sequence variations at the IRs/SSC junctions. Codon preference in the cpDNAs of the six *Hevea* species favors A/U endings, with ENc values all exceeding 45, suggesting a weak codon usage bias. Thirty-one optimal codons were pinpointed within the cpDNAs of these species. Phylogenetic analysis revealed that the *Hevea* genus is a monophyletic group, providing a theoretical foundation for genetic diversity studies and the development and utilization of specific genes within the *Hevea* chloroplast genome.

## Figures and Tables

**Table 1:** Synopsis of the cpDNAs of *Hevea* genus.

Species	GenBank Number	Genome Size (bp)	Genes	rRNA	tRNA	GC%
*H. benthamiana*	MT333859	161,124	91	8	36	35.72
*H. brasiliensis*	NC015308	161,191	92	8	37	35.73
*H. camargoana*	MN781109	161,291	91	8	36	35.72
*H. nitida*	MT413435	161,124	91	8	36	35.73
*H. pauciflora*	NC059798	161,123	91	8	36	35.75
*H. spruceana*	NC059799	161,093	91	8	36	35.72

## Data Availability

The original contributions presented in the study are included in the article/Supplementary Materials, further inquiries can be directed to the corresponding author.

## Supplementary Materials

The following supporting information can be downloaded at: https://www.mdpi.com/article/10.3390/genes16020201/s1; Supplementary File S1: Table S1. RSCU values of codons in the chloroplast genomes of six *Hevea* species. Supplementary File S2: Table S2. RSCU and ΔRSCU values in the high and low expression libraries in the chloroplast genomes of six *Hevea* species.
